# Vulnerability of peatland fires in bengkalis regency during the ENSO El nino phase using a machine learning approach

**DOI:** 10.1016/j.mex.2024.103128

**Published:** 2024-12-20

**Authors:** Fauziah, Lilik B. Prasetyo, Nonon Saribanon, Nur Hayati

**Affiliations:** a Department of Magister Technology Information, Faculty of Information and Communications Technology, Nasional University, Jl. Sawo Manila No. 61 RT.14/ RW.7, West Pejaten, Pasar Minggu, South Jakarta 12520, Indonesia; b Department of Forest Resources Conservation and Ecotourism, Faculty of Forestry and Environtment, IPB University, Jl. Raya Dramaga, Kampus IPB, Dramaga Bogor 16680 West Java, Indonesia; c Department of Agrotechnology, Faculty of Biology and Agriculture, Nasional University, Jl. Sawo Manila No. 61 RT.14/ RW.7, West Pejaten, Pasar Minggu, South Jakarta 12520, Indonesia; d Department of Information System, Faculty of Information and Communications Technology, Nasional University, Jl. Sawo Manila No. 61 RT.14/ RW.7, West Pejaten, Pasar Minggu, South Jakarta 12520, Indonesia

**Keywords:** Bengkalis regency, Burn area, Logistic regression, Peatland fire, Random forest, Random Forest, Logistic-Regression*g*

## Abstract

Peatland fires are increasingly becoming a concern as a recurring environmental issue in Indonesia, particularly along the east coast of Sumatra Island, in Bengkalis Regency. Therefore, the development of a peatland fire prediction model is necessary. This study aims to identify peatland fire vulnerability in Bengkalis Regency using burn area from MODIS 2019. The algorithm used are Random Forest (RF) and Logistic Regression (Log-Reg), with independent variables including physiography, peat physical characteristics, anthropogenic factors, climate, and NDMI. The total burned area in Bengkalis Regency in 2019 was 175.85 km², with Rupat District being the area with the largest burned area. The best model is RF that was able to predict peatland fires in Bengkalis Regency effectively, with achieving an AUC value of 0.972. The five main factors influencing peatland fires were road density, precipitation, drainage density, NDMI, and river density. The accuracy of RF reached 95.07%. The classification results indicated three levels of peatland fire vulnerability in Bengkalis Regency

• Non-Vulnerable: Areas classified as non-vulnerable are regions where the risk of peatland fires is minimal or non-existent.
• Low Vulnerability: These areas have a moderate risk of peatland fires.
• High Vulnerability: Areas with high vulnerability are the most susceptible to peatland fires.

## Graphical abstract

**Figure:**
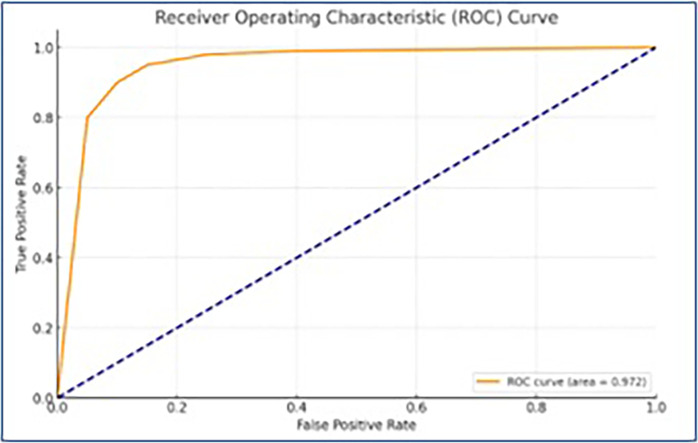

Specifications table

**Table:** 

Subject area:	Environmental Science
More specific subject area:	*Remote Sensing*
Name of your method:	Random Forest, Logistic-Regression*g*
Name and reference of original method:	Not applicable
Resource availability:	Not applicable

## Background

Sumatra, which contains the largest peatland area in Indonesia at 5.85 million ha, plays a critical role in mitigating climate change [1]. The peatlands in Sumatra are mainly located along the east coast, including Bengkalis Regency in Riau Province. Peatlands in Bengkalis cover 840,328 ha, accounting for approximately 66.50% of the total land area, and are consistently at risk of forest and land fires each year [2]. To reduce the harmful impacts of these fires, a peatland fire prediction model is essential, particularly for Bengkalis Regency. Two response variables commonly used to identify fires are burn area and hotspots. In this study,he the burn area variable is used to assess peatland fire vulnerability in Bengkalis. The burn area utilized comes from the MODIS satellite for the year 2019, with several predictor variables including physiography, peat physical characteristics, anthropogenic factors, climate, and NDMI [3]. To analyze vurnerabilty of peatland fires, we can use machine learning algorithm such Random Forest (RF) and Logistic Regression (Log-Reg). Some advantages of RF and Log-Reg are handle complex variables, resistance to overfitting, capacity to manage imbalanced data, and its model interpretability. The model's performance is evaluated based on the AUC value for both training and test data. In addition to model prediction, this study also examines the correlation between predictor variables and the occurrence of peatland fires in Bengkalis Regency [4]. Beside the predictor variable, El Niño Southern Oscillation (ENSO) can influences the incidence of fires in Indonesia by causing extended periods of hydrological drought. Therefore, evaluating the vulnerability of peatlands to fires and comprehending the factors driving this vulnerability are crucial for creating effective adaptation and mitigation strategies for peatlands [5]. Fires frequently occur in drained and degraded peatlands, In years of abnormally dry conditions, typically linked to El Niño, the peat dries out sufficiently to ignite [6]. In recent years, devastating wildfire events in the Sumatran peatlands have significantly increased greenhouse gas emissions. The ENSO El Niño influences fire occurrences in Indonesia by causing extended periods of hydrological drought. Therefore, it is crucial to evaluate the vulnerability of peatlands to fires and comprehend the factors contributing to this vulnerability in order to formulate effective adaptation and mitigation strategies for these ecosystems [7]. The transformation of peatlands in Indonesia has resulted in significant ecological and environmental damage. Changes in land use have turned these natural carbon sinks into ecosystems susceptible to drought and fire*.*

## Method details

### Study area

**Fig. 1:**
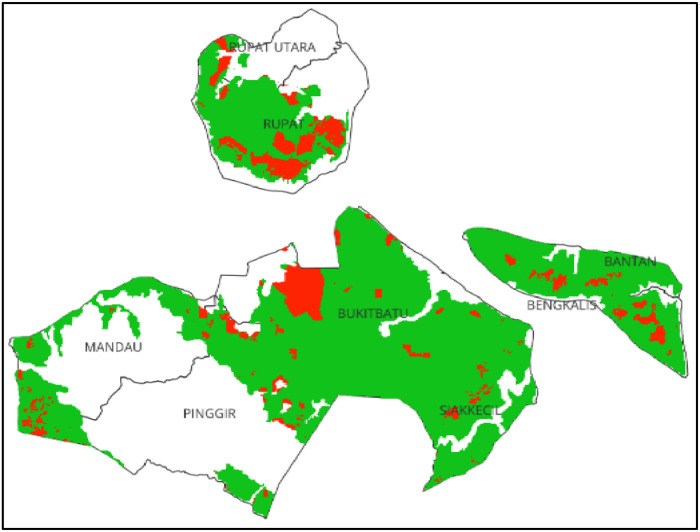
Distribution of burn area MODIS 2019 in peatland area of Bengkalis Regency.

This research was conducted in the administrative region of Bengkalis Regency, Riau Province (Fig. 1). Geographically, the regency is located on the eastern edge of Sumatra Island, covering an area of 7773.93 km². It is bordered by the Malacca Strait to the north, Siak Regency to the south, Meranti Islands Regency and Karimun Regency to the east, and Dumai City, Rokan Hilir Regency, and Rokan Hulu Regency to the west. Administratively, Bengkalis Regency consists of 11 sub-districts, but only 8 of them contain peatlands (Table 1). Bengkalis Regency's proximity to Malaysia and Singapore makes it a key location for stakeholders involved in the enforcement of the ASEAN Agreement on Transboundary Haze Pollution (AATHP) [15].

**Table 1:** Percentage of administrative area to peatland area in Bengkalis Regency.

District	Area (Ha)	Peatland Area (Ha)	%
Bantan	31,596.03	27,990.81	88.59
Bengkalis	58,952.14	53,305.5	90.42
Bukitbatu	203,686.53	172,858.13	84.86
Mandau	116,656.62	37,159.71	31.85
Pinggir	194,702.85	76,239.67	39.16
Rupat	107,473.3	67,817.63	63.10
Rupat Utara	42,953.29	11,846.17	27.58
Siakkecil	66,421.73	57,560.29	86.66

In Fig. 1 and Table 1 it can be seen that the largest peatland is in Bengkalis District (90.42%). Meanwhile, the total area of ​​peatland in Bengkalis Regency reaches 61.38% of the total area.

### Predictor Variables

Predictor variable of peatland fires are Physiographic factors (such as the density of rivers and canal networks), peat physical characteristics (including peat depth and decomposition type), variables related to human activities (such as the type of peatland use, cover, and road network density), and climate-related variables (such as monthly precipitation rate and moisture index).

The predictor variables used for the prediction process were previously processed using QGIS 3.32.1. Data sources used to produce:

– Physiographic variables were obtained from administrative maps, drainage networks and road networks at the **Badan Informasi Geospasial (BIG)**,
– Peat physical characteristics variables were obtained from **Fungsi Ekosistem Gambut (FEG)** map, depth and type of peat decomposition map (Wetlands International Indonesia (WII))
– Human activity variables were obtained from peatland distribution maps (**Kementerian Pertanian**), plantation forest concession maps (Global Forest Watch-World Resources Institute (GFW-WRI))
– Climate variables are obtained from monthly rainfall maps (CHIRPS) and humidity index maps (USGS-Earth Explore)

### Response variables

**Fig. 2:**
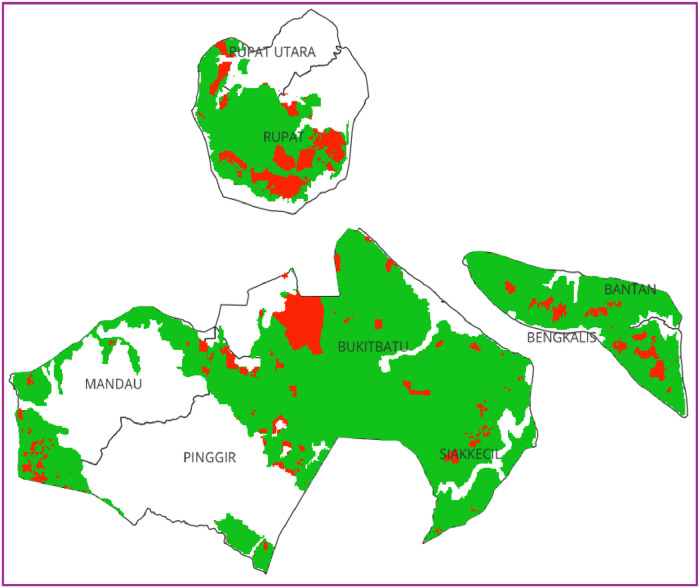
Burn area 2019 at peatland Bengkalis Regency.

The response variable used for the analysis of peatland fires in Bengkalis Regency is the burn area in 2019. The determination of the burn area year used is 2019 because in that year the last ENSO El Nino occurred until now. The results of the analysis used are by using two comparison algorithms, namely the Random Forest (RF) and Logistic Regression (Log-Reg) algorithms. The distribution of the burn area in 2019 can be seen in Fig. 2 below:

In 2019, Indonesia again experienced forest fires that burned peatlands covering an area of ​​238,000 Ha in the Sumatra and Kalimantan regions [8]. Just like in 2015, the El Nino phase hit Indonesia again with almost no rainfall in 2019 [9]. In 2019, the area burned in peatlands in Bengkalis Regency reached 27.44% of the total area of ​​peatlands. As seen in Table 2, the largest area burned was in Rupat District with a percentage of 14.33%. Although Rupat is not a district with the largest peat area in Bengkalis Regency, the area burned is the largest in the peatlands of Bengkalis Regency. This can be caused by the existence of peatland concession areas that have been converted into oil palm plantations and Hutan Tanaman Industri (HTI), which is 49,141.36 Ha [10]. So burning is often done intentionally.

**Table 2:** Percentage of peatland fires in Bengkalis Regency in 2019.

No	District	Burn area (Ha)	Peatland (Ha)	%
1	Bantan	925	27,990.81	3.30
2	Bengkalis	2773	53,305.50	5.20
3	Bukitbatu	741	172,858.13	0.43
4	Mandau	283	37,159.71	0.76
5	Pinggir	2515	76,239.67	3.30
6	Rupat	9719	67,817.63	14.33
7	Rupat Utara	0	11,846.17	0
8	Siakkecil	63	57,560.29	0.11

## Method validation

### Peatland fire prediction and influential predictor variables

Peatland fire prediction involves using various methods and models to anticipate the occurrence of fires in peatland ecosystems, which are particularly vulnerable due to their high organic content and moisture levels [11]. The entire process of model development and analysis in this study was conducted using R Studio software, which is an open-source programming tool that supports big data computing. The model development begins by dividing the collected data into two distinct datasets: the training dataset and the testing dataset. To achieve this, a stratified random sampling approach is applied, where the data is first categorized into six strata based on land use and land cover types (such as residential areas, vacant land, agricultural fields, plantations, forests, and shrubs). Then, within each stratum, the data is proportionally and randomly divided, with 80% allocated for the training dataset used in model development, while the remaining 20% is reserved as a testing dataset for model validation.

### Prediction

**Fig. 3:**
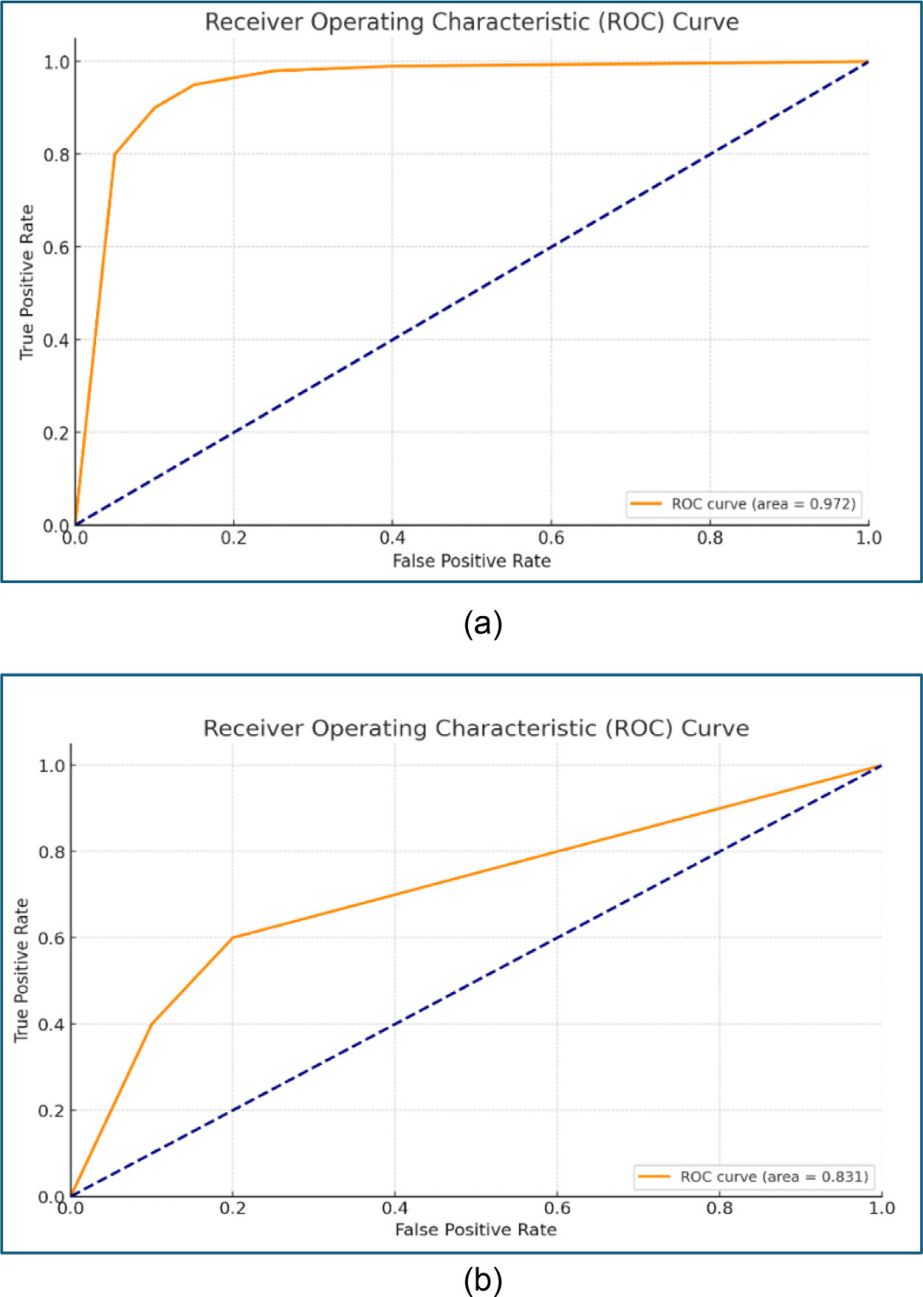
AUC of (a) Random Forest and (b) Logistic Regression.

The Random Forest (RF) model demonstrates an AUC of 0.972 and Logistic Regression (Log-reg) of 0.831 (Fig. 3). These results indicate that the RF model is effective in predicting peatland fires in Bengkalis Regency.

The impact of predictor variables on fire incidents in the peatland areas of Bengkalis Regency, as analyzed using the RF and Log-reg model are presented in Table 3 below:

**Table 3:** Influence of Predictor Variables of Random Forest and Logistic Regression.

	Random Forest	Logistic Regression
Drainage Density	2769,91	202.69
Road Density	5128,06	262.55
River Density	1332,09	-685.53
Water_Land Use	8,37	9.86
Forest_Land Use	350,84	9.52
Plantation_Land Use	285,71	9.21
Settlement_Land Use	8,06	5.72
Bush_Land Use	358,29	10.14
Land_Land Use	20,20	7.51
Depth_D1	121,39	-0.62
Depth_D2	214,60	0.64
Depth_D3	188,89	0.58
Depth_D4	407,00	NA
Type_H1a	52,61	-1.84
Type_H2a	203,19	-0.87
Type_H3a	201,49	-0.46
Type_H4a	388,68	NA
Type_S1a	56,18	NA
Type_S2c	10,94	-2.47
Type_S2a	184,72	NA
Type_S3a	252,66	NA
Precipitation	4536,90	0.03
NDMI	2209,72	-1.04

In Table 3, it is evident that five variables have the greatest influence on the RF model: Road Density (26.58%), Precipitation (23.52%), Drainage Density (14.36%), NDMI (11.45%), and River Density (6.91%). The remaining eighteen variables contribute relatively less than 5% each. Whereas Log-reg has seven variables exhibit negative values and five variables are not detected due to singularity, indicating that these twelve variables do not significantly influence peatland fires in Bengkalis Regency. The two variables that do have a significant impact on peatland fires are Road Density (50.64%) and Drainage Density (39.10%), while the remaining nine variables each contribute less than 5%.

**Fig. 4:**
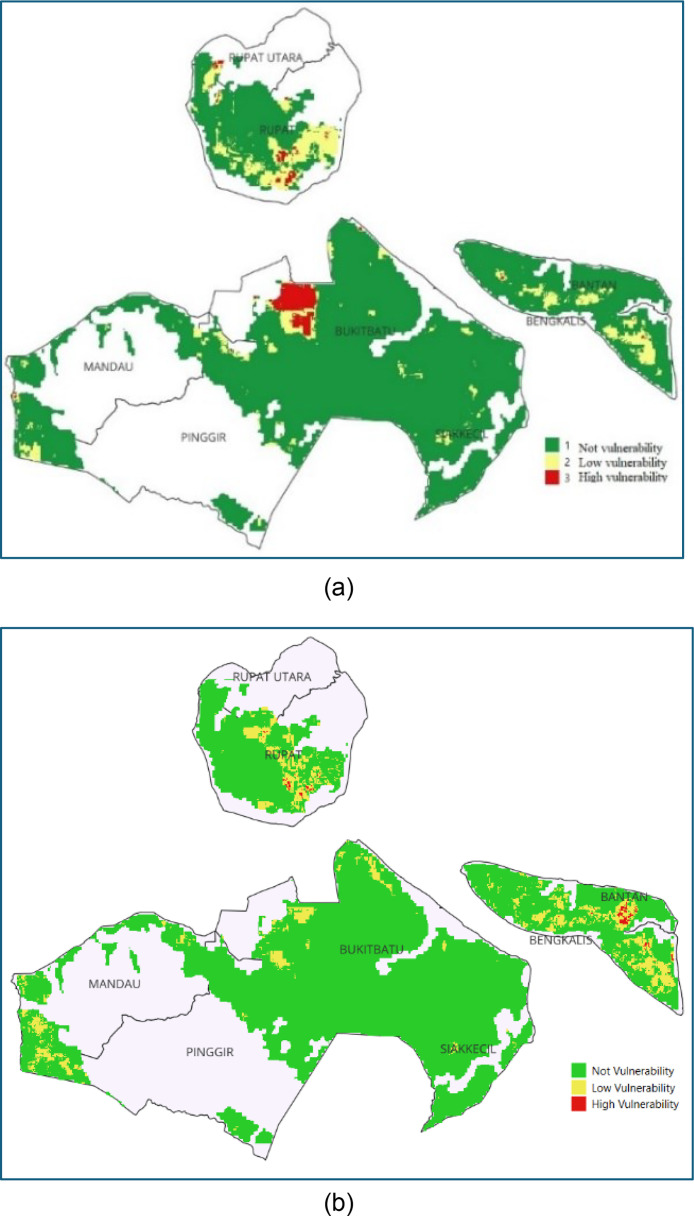
Peatland fire prediction map in Bengkalis Regency with Random Forest Algorithm (a) dan Logistic Regression (b).

The RF model predicts the probability of peatland fires with values ranging from 0 to 1 and achieves an accuracy rate of 95.07%. Wheares the Log-reg model predict achieves an accuracy rate of 85.16%. The classification results indicate three levels of peatland fire vulnerability in Bengkalis Regency: not vulnerable, low vulnerability, and high vulnerability (Fig. 4). The three districts identified as having the highest vulnerability to peatland fires in Bengkalis Regency are Bukitbatu, Rupat, and North Rupat for RF model and Rupat, Bengkalis, and Batan for Log-reg model.

## Limitations

Not applicable.

## Ethics statements

Not applicable.

## Supplementary material *and/or* additional information [OPTIONAL]

Not applicable.

## CRediT authorship contribution statement

**Fauziah:** Writing – review & editing, Supervision, Investigation. **Lilik B. Prasetyo:** Writing – review & editing, Supervision, Investigation. **Nonon Saribanon:** Writing – review & editing, Supervision. **Nur Hayati:** Conceptualization, Methodology, Software, Writing – original draft.

## Declaration of competing interest

The authors declare that they have no known competing financial interests or personal relationships that could have appeared to influence the work reported in this paper.
